# Adipokines—A Cohort Prospective Study in Children with Severe Burns

**DOI:** 10.3390/ijms25147630

**Published:** 2024-07-11

**Authors:** Silviu Constantin Badoiu, Dan Mircea Enescu, Raluca Tatar, Daniela Miricescu, Iulia-Ioana Stanescu-Spinu, Maria Greabu, Anca Magdalena Coricovac, Silvia Elena Badoiu, Viorel Jinga

**Affiliations:** 1 Department of Anatomy and Embriology, Faculty of Medicine, Carol Davila University of Medicine and Pharmacy, 8 Eroii Sanitari Blvd., 050474 Bucharest, Romania; silviu.badoiu@umfcd.ro; 2 Department of Plastic and Reconstructive Surgery, Life Memorial Hospital, 365 Grivitei Street, 010719 Bucharest, Romania; 3 Department of Plastic Reconstructive Surgery and Burns, Grigore Alexandrescu Clinical Emergency Hospital for Children, Faculty of Medicine, Carol Davila University of Medicine and Pharmacy, 37 Dionisie Lupu Street, 020021 Bucharest, Romania; dan.enescu@umfcd.ro (D.M.E.); raluca.tatar@umfcd.ro (R.T.); 4 Discipline of Biochemistry, Faculty of Dentistry, Carol Davila University of Medicine and Pharmacy, 8 Eroii Sanitari Blvd., 050474 Bucharest, Romania; maria.greabu@umfcd.ro; 5 Discipline of Physiology, Faculty of Dentistry, Carol Davila University of Medicine and Pharmacy, 8 Eroii Sanitari Blvd., 050474 Bucharest, Romania; 6 Discipline of Embriology, Faculty of Dentistry, Carol Davila University of Medicine and Pharmacy, 8 Eroii Sanitari Blvd., 050474 Bucharest, Romania; anca.coricovac@umfcd.ro; 7 Faculty of Medicine, Carol Davila University of Medicine and Pharmacy, 8 Eroii Sanitari Blvd., 050474 Bucharest, Romania; silvia.badoiu@stud.umfcd.ro; 8 Department of Urology, Carol Davila University of Medicine and Pharmacy, 8 Eroii Sanitari Blvd., 050474 Bucharest, Romania; viorel.jinga@umfcd.ro; 9 Academy of Romanian Scientists, 3 Ilfov, 050085 Bucharest, Romania

**Keywords:** adipokines, adiponectin, resistin, leptin, tumor necrosis factor-α (TNF-α), severe burns

## Abstract

Burns generate every year an important burden of morbidity, being a major global public health problem through prolonged hospitalization, complications, and increased mortality. This study’s purpose was to evaluate the serum levels of three adipokines and to establish significant correlations with other circulating molecules and with some clinical parameters. We evaluated 32 children with severe burns (over 25% total burned surface area—TBSA) at 48 h, day 10, and day 21 post burn, and 21 controls. The serum levels of adiponectin, resistin, leptin, tumor necrosis factor-α (TNF-α), plasminogen activator inhibitor-1 (PAI-1), and C-reactive protein (CRP) (among nine other biochemical parameters) were detected by Multiplex technique. Significant statistical differences were obtained for resistin and leptin compared to the control group, in different moments of measurements. Adiponectin serum levels presented statistically significant correlations with hot liquid mechanism of burn, the Revised Baux score, TBSA, resistin, PAI-1, CRP, TNF-α, and triglycerides (TGLs) serum levels. Resistin serum levels presented statistically significant correlations with adiponectin, CRP, PAI-1, leptin, and TNF-α. Additionally, we found statistically significant correlations between leptin serum levels and length of hospitalization, TNF-α, resistin, adiponectin, and PAI-1 serum levels. In severely burned children, adiponectin, resistin, and leptin specifically correlate with clinical parameters and with proteins involved in the systemic inflammatory response and the hypermetabolic response.

## 1. Introduction

As one of the most severe types of trauma [1], burns generate every year a huge burden of morbidity, being a major global public health problem [2]. Severe burns are often characterized by prolonged hospitalization, complications (multiple organ failure, sepsis), and increased rate of death [3]. Patients who survive from major burns often need serial surgical procedures (for contractures, scarring, etc.), permanent physical therapy (for physical sequelae and functional disabilities), and long-term psychological support (for permanent psychological issues) [4]. Better understanding of the inflammatory changes and of the metabolic alterations is needed for improvement of burns’ treatment in the acute phase and in the sequelae period.

The adipose tissue is one of the main organs involved in the systemic response to major trauma (including severe burns, extensive surgical procedures), sepsis, and malignancies [5,6]. In a previously published paper [7], we focused on the role of some of the acute-phase proteins in severe burns. In the present paper, our focus is upon three adipokines: adiponectin, resistin, and leptin.

Adiponectin is the most abundant adipokine [8,9]. It is produced by the fat tissue: subcutaneous fat [8], visceral fat [8], and bone marrow fat [9]. It has numerous effects, including an anti-inflammatory effect [8,10], stimulation of thermogenesis [10], stimulation of fatty acids’ oxidation in liver and skeletal muscle [9,10], stimulation of lipolysis [9,10], induction of browning of the white adipose tissue [9], inhibition of gluconeogenesis and enhancement of fatty acid biosynthesis in the liver [10], and enhancement of glucose uptake in the skeletal muscle [10]. 

Resistin is an adipokine produced by several cells and tissues: fat tissue, skeletal muscle, intestinal epithelium, monocytes, and astrocytes [8,9,11]. It has a pro-inflammatory effect, augmenting the expression of cytokines like interleukin-6 (IL-6) and tumor necrosis factor-α (TNF-α/TNF-alpha) in the adipose tissue [12], being involved in the development and the persistency of an inflammatory status in obesity, type 2 diabetes mellitus [13], metabolic syndrome [13], cardiovascular diseases [14,15], and sepsis [12]. Resistin also has an important role in immunity, as it directly activates the complement system [16], has a chemotactic effect upon leukocytes [17], and contributes to adhesion molecules expression (like TNF-a and IL-6) [18]. 

Leptin is an adipokine secreted mainly by the white adipose tissue but also by brown adipose tissue, skeletal muscle, stomach, breast, ovaries, and placenta (during pregnancy) [19,20,21,22,23]. It contributes to the balance between food intake and energy expenditure [24], being one of the main regulators of the white adipose tissue mass [23]. It acts upon specific receptors in the hypothalamus and in peripheral tissues (such as white adipose tissue, heart, gut, liver, pancreas). In the hypothalamus, leptin acts at the level of the Arcuate Nucleus and influences food intake through inhibition of orexigenic peptides [25] and stimulation of anorexigenic neuropeptides [26]. The Arcuate Nucleus’ neurons are connected with the nuclei in the lateral hypothalamic area and paraventricular area, which are controlling the energy balance [23]. In the periphery, leptin has endocrine, paracrine, and autocrine actions, and is involved in the regulation of energy expenditure and metabolism, together with other hormones: glucocorticoids, glucagon, insulin, growth hormone [27]. In the adipose tissue, leptin stimulates lipolysis and fatty acid’s oxidation, and inhibits lipogenesis and insulin-dependent glucose uptake [28]. Leptin acts upon fat tissue directly, through interaction with its specific receptors [29], and indirectly too, through the sympathetic system [30,31]. 

TNF-α is a cytokine and a member of the TNF superfamily of proteins [32]. It is mainly produced by monocytes/activated macrophages, lymphocytes, but also by the adipose tissue, being considered from this point of view, an adipokine [33]. TNF-α exists in two forms: a transmembrane form (mTNF-α) and a soluble form (sTNF-α) [34]. 

TNF-α acts upon two types of receptors: (a) TNFR1—expressed in the majority of cell types—their stimulation being responsible for the pro-inflammatory and pro-apoptotic signaling; (b) TNFR2—expressed in endothelial, epithelial, and several types of immune cells—their stimulation being responsible for the anti-inflammatory, cell-proliferation, and wound-healing signaling [35,36].

The targeted molecules in the present study were: adiponectin, resistin, and leptin. We determined their serum levels during the first 3 weeks post burn. Applying specifical methods of statistical analysis, we looked for any significant correlations with markers of the systemic inflammatory response (such as acute-phase proteins), markers of the hypermetabolic state (such as triacylglycerols), with common clinical parameters (such as burn mechanism, TBSA, hospitalization) and with one of the popular prognostic indexes in burns (the Revised Baux score). Finally, we wanted to see if any adipokine from the analyzed group had any value as a predictive marker of the evolution of children with burns exceeding 25% TBSA (total burned surface area).

Adipose tissue and its hormones are important in the systemic response to severe trauma, sepsis, and, generally, in critically ill patients. Adiponectin, leptin, and resistin have a major impact upon the hypermetabolic state, systemic inflammation, immune status, and the healing process, in severe burns. These are proven facts in adult burns but not completely proven in pediatric burns. That is why we consider our study to be relevant for clarifying the role of the abovementioned adipokines in the evolution of severely burned children.

## 2. Results

Table 1 illustrates the characteristics: study group–control group. The study group consisted of 32 burned children (60.4%), and the control group consisted of 21 non-burned children (39,6%). Fisher test indicated similar gender frequencies in the groups (*p* = 0.089). Applying Mann–Whitney U Test, significant differences of age were identified in the study versus control groups: median = 3 years, IQR = 2–10 years (in months: median = 39.5, IQR = 26–122.5), respectively, median = 14 years, IQR = 12–16 years (*p* < 0.001).

Clinical characterization of the study group: TBSA-median = 35% (IQR = 30–45%), the percentage of deep burn (grade IIB/III)-median = 20%, IQR = 16.5–33%, time elapsed from burn trauma to hospital admission-median = 8 h (IQR = 4–9.5 h), patients with smoke inhalation injury =11 (34.4%), patients with mechanical ventilation = 25 (78.1%), Revised- Baux Score median = 50 (IQR = 31–64), burn injury mechanism: hot liquids = 15 (46.9%), flames = 13 (40.6%), deceased patients = 2 (6.3%)

Data from Figure 1 show the values of adipokines in the target group. According to the Shapiro–Wilk test, distribution of the analyzed variables was non-parametric across most of the results (*p* < 0.05). Significant differences between measurements were revealed only for resistin measurements in conformity to the Friedman test (*p* = 0.006). Also, post hoc tests concludes that measured serum levels at T2 (median = 445.6, IQR = 317.62–1012) were significantly higher than those measured at T1 (median = 335.56, IQR = 95.25–622.3) (*p* = 0.012) and higher than those measured at T3 (median = 267.5, IQR = 146.2–450.13) (*p* = 0.026). The evolutions of adiponectin (*p* = 0.104) and leptin (*p* = 0.097) levels were not statistically significant, while noticeable was a tendency towards statistical significance for leptin measurements (*p* = 0.097) towards a lower value at T2 versus T1, while the evolution of adiponectin shows a tendency towards higher values at T2 and T3 versus T1.

Data from Figure 2 show the comparison of analyzed adipokines between the study and control group. Across most of the measurements in both groups, distribution of the analyzed values was non-parametric in conformity to the Shapiro–Wilk test (*p* < 0.05). Differences between analyzed groups were statistically significant only in the case of resistin (*p* < 0.001) and leptin (*p* = 0.041) measurements at T2 showing higher values of resistin in the study group versus control group (median = 445.6, IQR = 316.8–915.89 vs. median = 188.38, IQR = 87.02–274.18) and lower values of leptin in the study group versus control group (median = 1357.4, IQR = 610.2–3550.5 vs. median = 3476.16, IQR = 1171.2–10,705.8). A tendency towards statistical significance was observed in the case of observed differences between resistin measurements at T1 (*p* = 0.052) and at T3 (*p* = 0.053) showing potential higher values of resistin in the study group in comparison to the control group.

**Figure 3:**
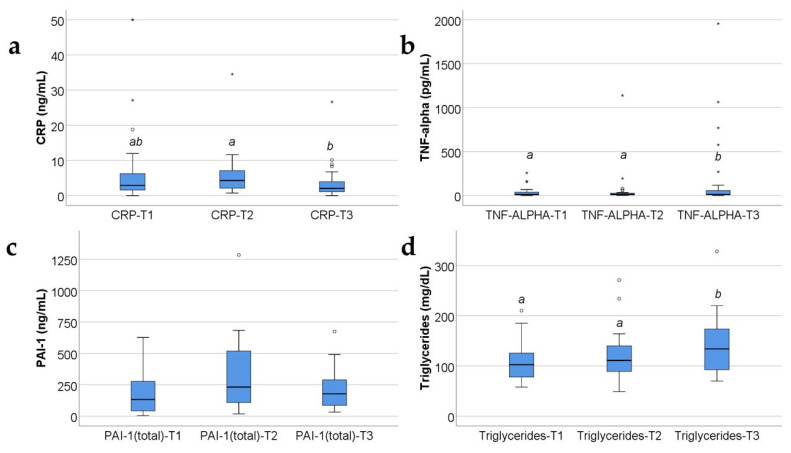
The box-plot graphic illustration for acute-phase inflammation proteins (CRP (**a**), TNF-α (**b**), PAI-1 (**c**)) and for triacylglycerols (Triglycerides) (**d**) in the burned children group. The values of the studied molecules are medians with interquartile ranges. Concentrations measured in ng/mL for CRP and PAI-1, pg/mL for TNF-α and mg/dl for triglycerides. Friedman’s tests with Dunn–Bonferroni post hoc tests were applied for statistical analysis. For PAI-1, no statistically significant differences between measurements (*p* = 0.055) were identified. A significant difference between measurements is marked by unidentical lowercase letters. In the box-plot graphical representation, the ° symbol marks an outlier for a measurement < 1st quartile-1,5IQR or a measurement > 3rd quartile +1,5IQR. The * symbol marks an extreme outlier, for a measurement < 1st quartile-1,5IQR or a measurement > 3rd quartile +1,5IQR.

Data from Figure 3 highlight the variations of common serum molecules in the study group. According to the Shapiro–Wilk test, the distribution of the analyzed molecules was non-parametric across most of the measurements (*p* < 0.05). Significant differences between registered values were identified for CRP (*p* = 0.014), TNF-α (*p* = 0.009), and triglycerides (*p* = 0.002), according to the Friedman tests. The data from post hoc tests highlight the following results:

CRP values were significantly higher at T2 (median = 4.26, IQR = 1.73–7.21) than at T3 (median = 2.02, IQR = 0.93–4.12) (*p* = 0.012);

TNF-α values were significantly higher at T3 (median = 13.35, IQR = 8.75–71.86) than at T2 (median = 12.22, IQR = 7.65–31.1) (*p* = 0.029) or at T1 (median = 13.24, IQR = 6.24–42.6) (*p* = 0.019);

Triglyceride values were significantly higher at T3 (median = 134, IQR = 92.25–173.75) than at T2 (median = 111, IQR = 88.5–141) (*p* = 0.040) or at T1 (median = 102.5, IQR = 78–125.75) (*p* = 0.003);

Also observed was a tendency towards statistical significance in the case of PAI-1 measurements evolution (*p* = 0.055), indicating potentially higher values at T2 than at T1 or T3.

**Figure 4:**
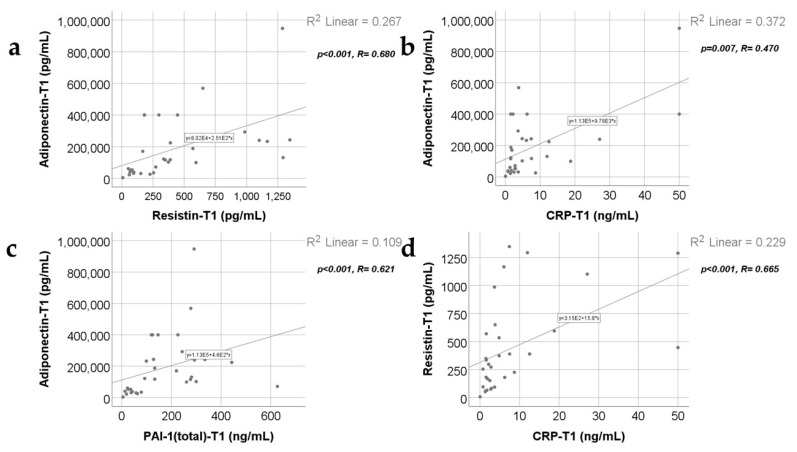
Correlations between adiponectin value at T1 and resistin value at T1 (**a**), CRP level at 48 h (**b**), and PAI-1 levels at T1 (48 h) (**c**), along with the correlation between resistin levels at T1 and CRP levels at T1 (**d**).

**Figure 5:**
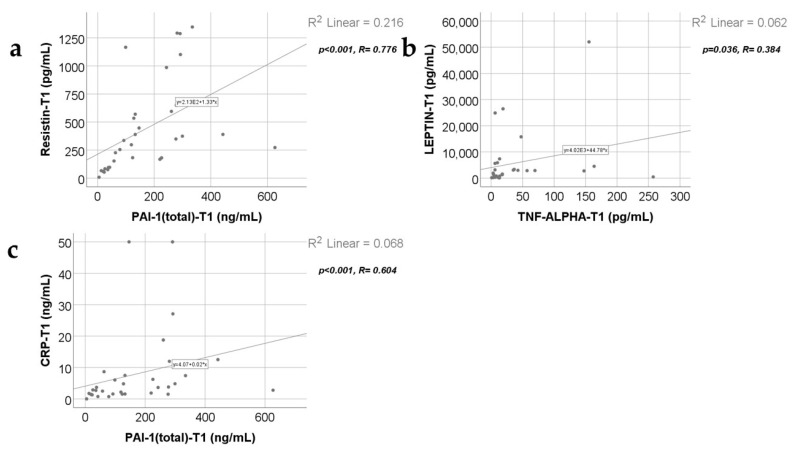
Correlations between resistin levels at T1 and PAI-1 levels at T1 (**a**), leptin value at T1 and TNF-α at T1 (**b**), and CRP value at T1 and PAI-1 value at T1 (**c**).

**Figure 6:**
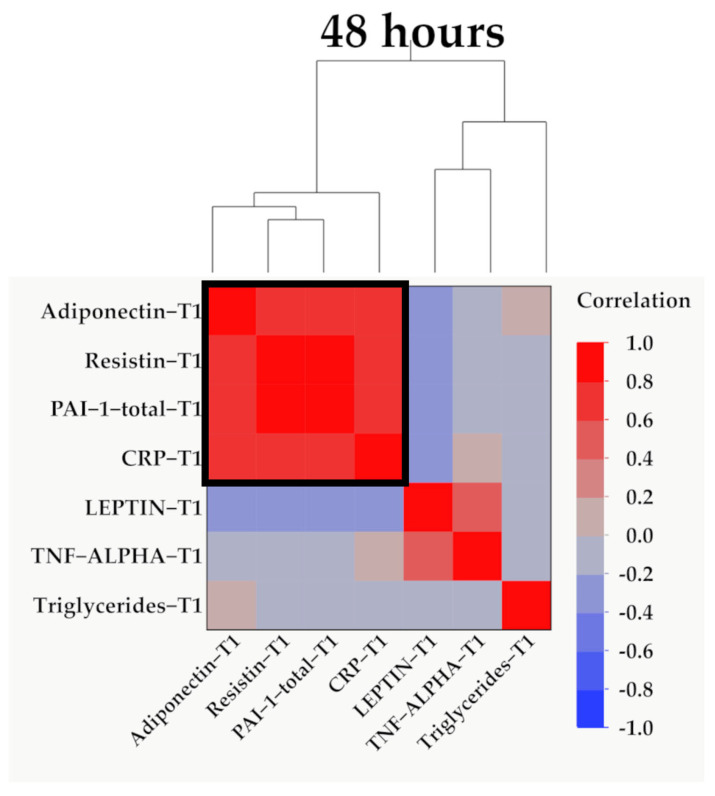
Hierarchical clustering of adipokines, inflammatory parameters, and triglyceride values at 48 h (T1). Variables were transformed to base10 logarithm values for data normalization. Data show the clustering of Pearson’s correlation coefficients, where a cluster between adiponectin, resistin, PAI-1, and CRP is indicated by the outlined box.

Data from Figure 4, Figure 5 and Figure 6 show the correlations for analyzed parameters at T1. According to the Shapiro–Wilk test, the distribution of the measured values was non-parametric (*p* < 0.05). The following correlations were observed to be significant positive correlations with moderate/high power according to the Spearman’s rho coefficients between the following parameters (*p* < 0.05):

Adiponectin and resistin (*p* < 0.001, R = 0.680), CRP (*p* = 0.007, R = 0.470) or PAI-1 (*p* < 0.001, R = 0.621);

Resistin and CRP (*p* < 0.001, R = 0.665) or PAI-1 (*p* < 0.001, R = 0.776);

TNF-α and leptin (*p* = 0.036, R = 0.384);

CRP and PAI-1 (*p* < 0.001, R = 0.604).

**Figure 7:**
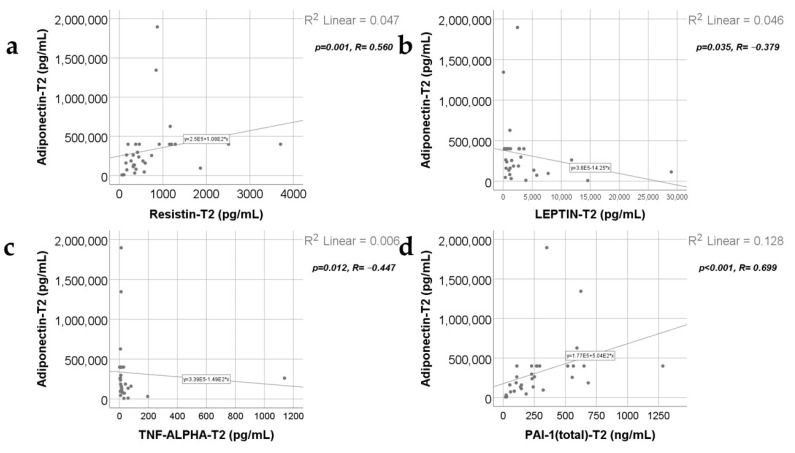
Correlations between adiponectin levels at T2 and resistin levels at T2 (**a**), leptin value at T2 (**b**), TNF-α value at T2 (**c**), and PAI-1 levels at T2 (**d**).

**Figure 8:**
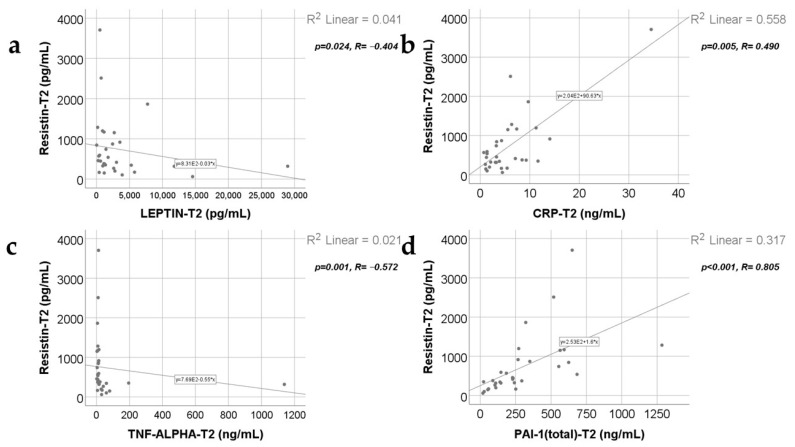
Correlations between resistin levels at T2 and leptin levels at T2 (**a**), CRP value at T2 (**b**), TNF-α value at T2 (**c**), and PAI-1 levels at T2 (**d**).

**Figure 9:**
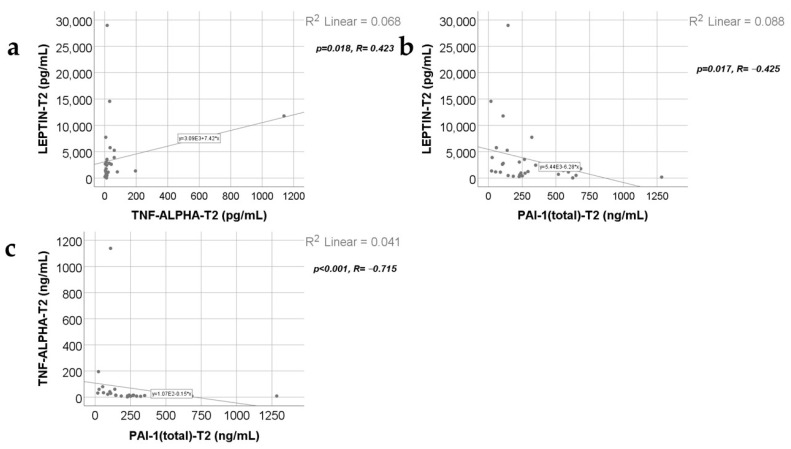
Correlations between leptin levels at T2 and TNF-α levels at T2 (**a**), PAI-1 value at T2 (**b**), and the correlation between TNF-α and PAI-1 values at T2 (**c**).

**Figure 10:**
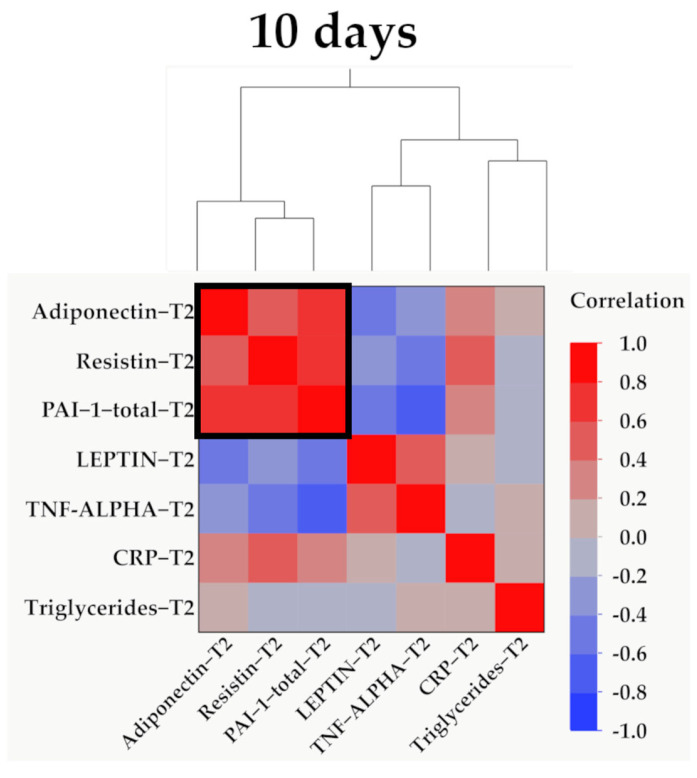
Hierarchical clustering of adipokines, inflammatory parameters, and triglyceride values at 10 days (T2). Variables were transformed to base10 logarithm values for data normalization. Data show the clustering of Pearson’s correlation coefficients, where a cluster between adiponectin, resistin, and PAI-1 is indicated by the outlined box.

Data from Figure 7, Figure 8, Figure 9 and Figure 10 show the correlations for analyzed parameters at T2. According to the Shapiro–Wilk test, the distribution of the values was non-parametric (*p* < 0.05). The following correlations were observed to be significant positive/negative correlations with moderate/high power according to the Spearman’s rho coefficients between the following parameters (*p* < 0.05):

- Adiponectin and resistin (*p* = 0.001, R = 0.560), leptin (*p* = 0.035, R = −0.379), TNF-α (*p* = 0.012, R = −0.447) or PAI-1 (*p* < 0.001, R = 0.699);
- Resistin and leptin (*p* = 0.024, R = −0.404), CRP (*p* = 0.005, R = 0.490), TNF-α (*p* = 0.001, R = −0.572) or PAI-1 (*p* < 0.001, R = 0.805);
- TNF-α and leptin (*p* = 0.018, R = 0.423);
- PAI-1 and leptin (*p* = 0.017, R = −0.425);
- TNF-α and PAI-1 (*p* < 0.001, R = −0.715).

**Figure 11:**
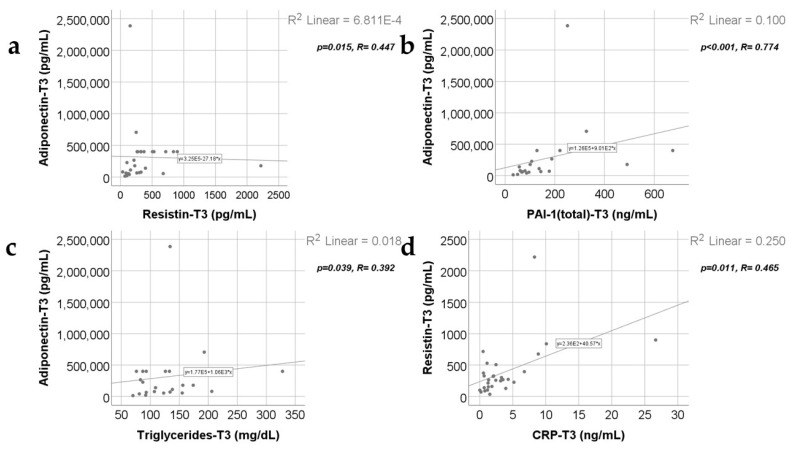
Correlations between adiponectin values at T3 (21 days) and resistin levels at T3 (21 days) (**a**), PAI-1 levels at T3 (21 days) (**b**), triglycerides value at T3 (**c**), along with the correlation between resistin and CRP values at T3 (**d**).

**Figure 12:**
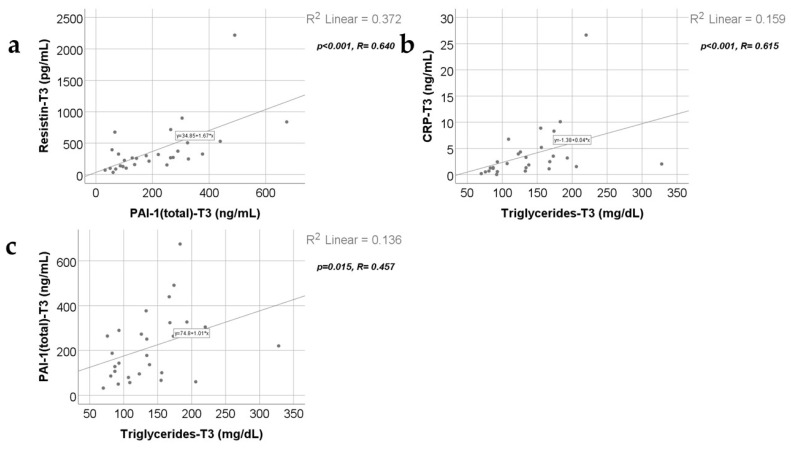
Correlation between resistin levels at T3 (21 days) and PAI-1 levels at T3 (21 days) (**a**), along with the correlations between triglycerides value at T3 and CRP value at T3 (**b**) and PAI-1 values at T3 (**c**).

**Figure 13:**
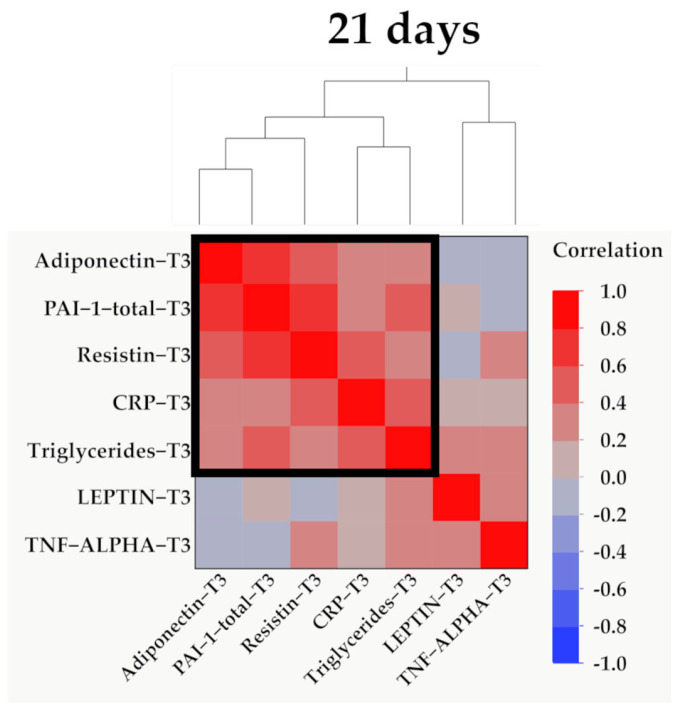
Hierarchical clustering of adipokines, inflammatory parameters, and triglyceride values at 21 days (T3). Variables were transformed to base10 logarithm values for data normalization. Data show the clustering of Pearson’s correlation coefficients, where a cluster between adiponectin, PAI-1, resistin, CRP, and triglycerides is indicated by the outlined box.

Data from Figure 11, Figure 12 and Figure 13 show the correlations for analyzed parameters at T3. According to the Shapiro–Wilk test, the distribution of the values was non-parametric (*p* < 0.05). The following correlations were observed to be significant positive/negative correlations with moderate/high power according to the Spearman’s rho coefficients between the following parameters (*p* < 0.05):

- Adiponectin and resistin (*p* = 0.015, R = 0.447), PAI-1 (*p* < 0.001, R = 0.774) or triglycerides (*p* = 0.039, R = 0.392);
- Resistin and CRP (*p* = 0.011, R = 0.465) or PAI-1 (*p* < 0.001, R = 0.640);
- Triglycerides and CRP (*p* < 0.001, R = 0.615) or PAI-1 (*p* = 0.015, R = 0.457).

Data from Figure 14 show the correlation between adiponectin value at T1 and TBSA. According to the Shapiro–Wilk test, the distribution of TBSA was non-parametric (*p* = 0.003). We found a significant and negative correlation (*p* = 0.018, R = −0.416), which highlights that patients with augmented measurements of TBSA were statistically significantly more associated with low levels for adiponectin at T1. while patients with decreased values of TBSA were statistically significantly more associated with augmented levels of adiponectin.

Data from Figure 15 show the correlations between leptin values and the hospitalization period. According to the Shapiro–Wilk test, the distribution of the hospitalization period values was non-parametric (*p* = 0.003). Statistically significant and negative correlations were found for the leptin value at T2 (*p* = 0.021, R = −0.412) or at T3 (*p* = 0.035, R = −0.393), which demonstrates that patients with longer hospitalization periods were statistically significantly more associated with low levels for leptin at T2/T3 while patients with shorter hospitalization periods were statistically significantly more associated with augmented leptin levels at T2/T3.

Data from Figure 16 show the comparison of adipokines between patients according to the mechanism of burn injury. According to the Shapiro–Wilk test, the distribution of the values was non-parametric across most of the measurements in both groups (*p* < 0.05). According to the Mann–Whitney U tests, the differences between analyzed groups were statistically not significant for most of the parameters (*p* > 0.05), except for the adiponectin value at T1 (*p* = 0.019), which was significantly higher for patients with hot liquid burns (median = 239,812, IQR = 60,634–400,000) in comparison to patients with flame burns (median = 71,495, IQR = 27,929–177,394).

Data from Figure 17 show the correlation between adiponectin value at T1 and R-Baux Score. According to the Shapiro–Wilk test, the distribution of the score was non-parametric (*p* = 0.035). We found a statistically significant and negative correlation (*p* = 0.010, R = −0.456); this points out that patients with high adiponectin serum levels were statistically significant more associated with low values of R-Baux Score and vice versa.

## 3. Discussion

In this section, we address only the statistically significant (positive/negative) correlations between the studied adipokines and clinical/biochemical parameters.

### 3.1. Adiponectin

**Figure 1:**
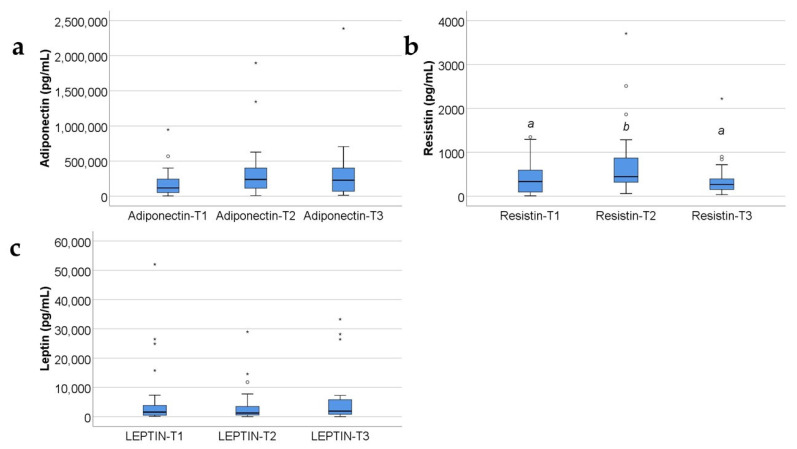
The box-plot representation for adiponectin (**a**), resistin (**b**), and leptin (**c**) variations in the target group (serum levels expressed in pg/mL). The values of the studied molecules are medians with interquartile ranges. Friedman’s tests with Dunn–Bonferroni post hoc tests were applied for statistical analysis. A significant difference between measurements is marked by unidentical lowercase letters. No statistically significant differences were observed for adiponectin (*p* = 0.104), nor for leptin (*p* = 0.097). In the box-plot graphical representation, the ° symbol marks an outlier for a measurement < 1st quartile-1,5IQR or a measurement > 3rd quartile +1,5IQR. The * symbol marks an extreme outlier, for a measurement < 1st quartile-1,5IQR or a measurement > 3rd quartile +1,5IQR.

In our study group (Table S1 and Figure 1a), adiponectin serum levels decrease at 48 h, showing the severity of trauma, and increase at 10 and 21 days, correlating with a good prognosis. Our results confirm previous studies’ reports about: (a) the precocious decreased levels of adiponectin as a hallmark in severe trauma (including severe burns), in sepsis and in critical illness; (b) the progressive increase in adiponectin serum levels as marker of a good prognosis of the critically ill patients (including any type of severe trauma, severe burns, and sepsis) [8,37,38]. A clinical application could be the early determination of adiponectin serum level and its usage as a prognostic marker in severely burned children. Following the dynamics of adiponectin serum levels might show us the tendency towards improvement or towards worsening of the prognosis.

Adiponectin serum levels, in our study, presented a statistically significant and negative correlation (Table S12 and Figure 17) with the R-Baux score [39]. The clinical significance of our observation is that patients with lower adiponectin levels at 48 h post burn have a higher R-Baux score, which means a worse prognosis. This corresponds to other studies that reported severity of burn and worse prognosis to be characterized by decreased adiponectin levels early post burn [8].

According to the burn injury mechanism (hot liquids versus flames), in our study, adiponectin serum level at T1 correlated statistically significantly and positively with the hot liquid burn mechanism (Table S11 and Figure 16a); the clinical significance was that patients with scalds presented higher levels of serum adiponectin at 48 h and a better prognosis, unlike patients with burns produced by flames, who had a lower adiponectin serum level and worse prognosis.

We found no statistically significant correlation between adiponectin serum values and the hospitalization length (Table S10). But we discovered a negative, statistically significant correlation between TBSA and adiponectin level at T1 (Table S9 and Figure 14); the clinical significance was that low adiponectin serum levels at 48 h post burn strongly correlated with larger TBSA (which is a predictor of burn severity [40] and an independent parameter associated with in-hospital mortality [41]).

At 48 h, 10 days, and 21 days post burn, adiponectin serum levels correlate positively with resistin (Tables S6–S8 and Figure 4a and Figure 7a, Table S11) and with PAI-1 serum levels (Tables S6–S8 and Figure 4c, Figure 7d and Figure 11b). The practical conclusions resulting from these correlations are that:

a. adiponectin–resistin interplay influences the metabolic profile in severely burned patients (as these adipokines have opposite actions upon the systemic inflammatory response and upon metabolism);
b. adiponectin–PAI-1 interplay influences the systemic inflammatory response, the peripheral insulin sensitivity, and the pro/anti-coagulant status in severely burned children (adiponectin is anti-inflammatory and decreases insulin resistance, meanwhile PAI-1 is pro-inflammatory, increases insulin resistance, and has antifibrinolytic and procoagulant action) [42,43].

Adiponectin serum levels correlate positively with CRP serum levels (Table S6 and Figure 4b) at 48 h. The practical conclusion is that an anti-inflammatory adipokine correlates significantly with a marker of systemic inflammation in severely burned children. So, determining the adiponectin serum levels might be used as an indicator of the severity of the systemic inflammatory response in severe burns. Previous studies reported a strong and negative correlation between adiponectin serum levels and CRP in healthy individuals [44] but also in patients with obesity, diabetes mellitus type 2, and metabolic syndrome [45].

Adiponectin serum levels negatively correlate with TNF-α serum levels (Table S7 and Figure 7c), unlike other studies where the correlation reported was positive [46,47]. It appears to be a reciprocal influence: adiponectin stimulates the production and release of TNF-α (and IL-6) in adipose tissue through extracellularly regulated kinase (ERK) and nuclear factor-kappa B (NF-κB) signaling [46], and TNF-α regulates the multimerization and secretion of adiponectin [47]. 

In severely burned children, increased adiponectin serum levels correlate with increased leptin serum levels (Table S7 and Figure 7b), which is different than the negative correlation between adiponectin and leptin that was reported in healthy individuals, but also in patients with obesity and metabolic syndrome [48].

Interestingly, adiponectin was the only adipokine in our study that showed a statistically significant and positive correlation with TGL at 21 days post burn (Table S8 and Figure 11c), reflecting the increased levels of lipolysis in adipocytes [49,50,51]. We consider that, in clinical practice, following the serum levels of adiponectin and of TGL might help us in evaluating the intensity of the hypermetabolic response in severe burns. 

### 3.2. Resistin

In our study, resistin serum levels increased at 48 h and even more at 10 days; the afterwards decrease towards 21 days heralded a good evolution of the patients (Table S1 and Figure 1b). Our study results confirm and refine other studies’ reports about resistin serum levels in critically ill patients (including those with severe burns, sepsis, trauma) [6,8].

Our results did not show any statistically significant correlation between resistin serum levels and the Revised Baux score (Table S12), although other studies reported a statistically significant and positive correlation between resistin serum levels at 7 days and other scores, like SOFA score (Sequential Organ Failure Assessment) [6].

We did not find any statistically significant correlation between resistin serum values and the burn injury mechanism (Table S11 and Figure 16b), the length of the hospitalization (Table S10), nor the TBSA (Table S9). TBSA (together with the burn depth) is one of the main determinants of prognosis in burns. Somehow, our results disagree with other studies that affirmed a positive correlation between resistin serum levels and the gravity of prognosis in severe burns [6,52].

Increased resistin serum levels correlated with increased CRP serum levels at 48 h, 10 days, and 21 days (Tables S6–S8 and Figure 4d, Figure 8b and Figure 11d). Our finding agrees with other studies describing, in severe burns, a positive correlation between this proinflammatory adipokine and this marker of systemic inflammation [6,8]. Resistin was considered to form a network with inflammatory cytokines in severe burns [6]. We consider that following the resistin serum levels might be helpful for evaluating the intensity of the systemic inflammatory response.

We found a positive correlation between resistin serum levels and PAI-1 serum levels (Tables S6–S8 and Figure 5a, Figure 8d and Figure 11b) in severely burned children, just like other studies reported the same type of correlation in metabolic syndrome and myocardial infarction [53]. PAI-1, in addition to its role in thrombus formation, is a marker of systemic inflammation [54], and its expression in adipocytes is regulated by resistin through AKT phosphorylation [55,56].

Unlike other studies, reporting a positive correlation of resistin serum levels with TNF-α serum levels in diseases with systemic inflammatory response or subclinical inflammation [57,58,59,60], in our study, increased resistin serum levels correlated with decreased TNF-α serum levels at 10 days (Table S7 and Figure 8c). Some studies reported resistin to induce in macrophages the secretion of some pro-inflammatory cytokines (among which there is TNF-α), by NF-κB-dependent pathway [61].

**Figure 2:**
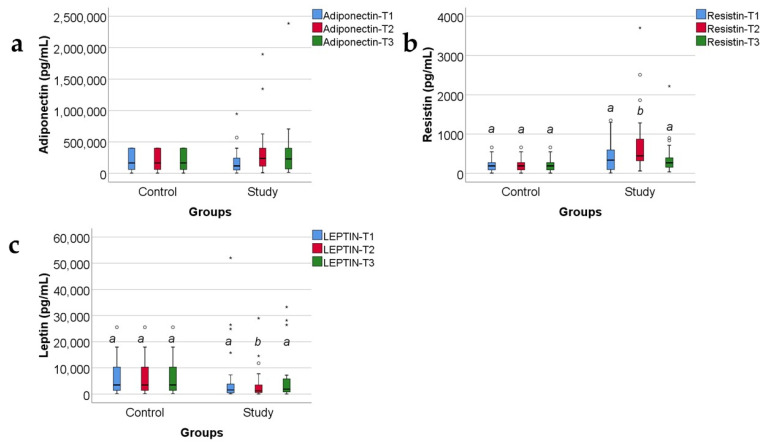
Concentrations, in pg/mL, of the three adipokines (adiponectin (**a**), resistin (**b**), and leptin (**c**)) in the study group and the control group, expressed as medians with IQR. Mann–Whitney U tests for statistical analysis. A significant difference between measurements is marked by unidentical lowercase letters. No statistically significant differences were observed for adiponectin, nor for leptin (*p* > 0.05). In the box-plot graphical representation, the ° symbol marks an outlier for a measurement < 1st quartile-1,5IQR or a measurement > 3rd quartile +1,5IQR. The * symbol marks an extreme outlier, for a measurement < 1st quartile-1,5IQR or a measurement > 3rd quartile +1,5IQR.

**Figure 14:**
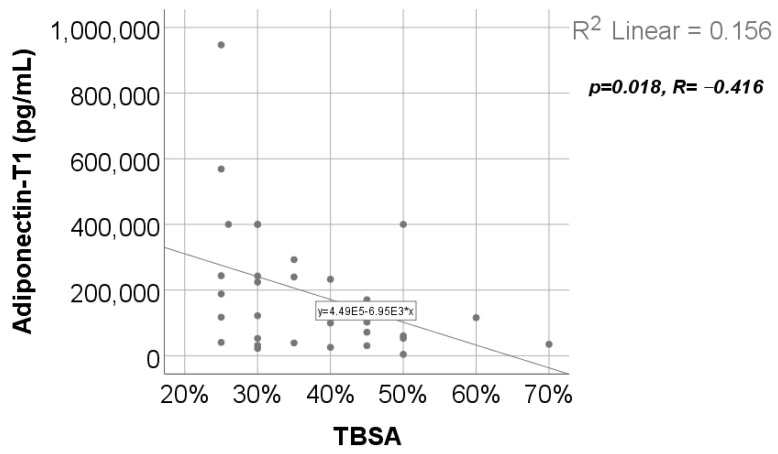
Correlation between TBSA and adiponectin value at T1.

In our study, increased resistin serum levels significantly correlate with decreased leptin serum levels (Table S7 and Figure 8a). Although resistin and leptin are both proinflammatory adipokines, in our study, their serum levels variate in opposite directions at 10 days post burn: resistin increases and leptin decreases (Figure 1b,c and Figure 2b,c). Other studies reported a positive correlation between resistin and leptin serum levels in metabolic syndrome, obesity, and diabetes mellitus type 2 [62]. In severe burns, several studies reported increased resistin and leptin serum levels, with/without a statistically significant correlation between them [6,52].

### 3.3. Leptin

Older studies reported leptin serum levels to remain within normal ranges, in patients with burns, excepting the cases complicated with hypovolemic shock [63]. Some studies reported that severe trauma lowered the leptin serum levels, and the favorable evolution of the patients had been announced by an increase in serum leptin [64]. Indeed, at 10 days, in our study, the leptin serum levels were significantly lower in the burned children when compared with the control group (Table S3 and Figure 2c). Other studies reported that leptin increases in severe burns, but non-survivors and patients with sepsis presented lower leptin serum levels when compared with severely burned patients who had no sepsis and had survived [65]. In critically ill patients with sepsis, another study reported that “lower serum leptin at enrollment and day 7 was independently associated with 28-day mortality”, and leptin levels were higher in survivors than in non-survivors [66]. This is concordant with our study results, in which all survivors had an increase in serum leptin levels at 21 days (Table S1 and Figure 1c). We consider that following the dynamics of leptin serum levels might show us the tendency towards amelioration or towards worsening of the prognosis.

Leptin serum levels did not correlate statistically with the Revised Baux Score (Table S12), with the burn injury mechanism: hot liquids versus flames (Table S11 and Figure 16c), nor with the TBSA (Table S9). Other studies reported higher leptin serum levels in patients with longer hospitalization [65] and in burned patients, but only when TBSA exceeded 30% [65,67].

**Figure 15:**
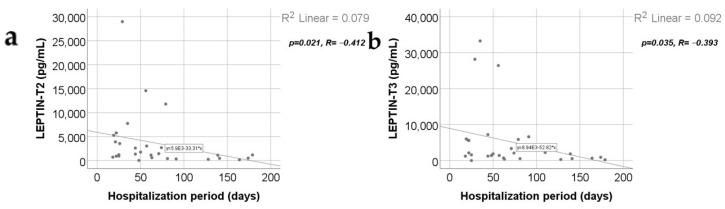
Correlations between hospitalization period and leptin value at T2 (**a**) and T3 (**b**).

**Figure 16:**
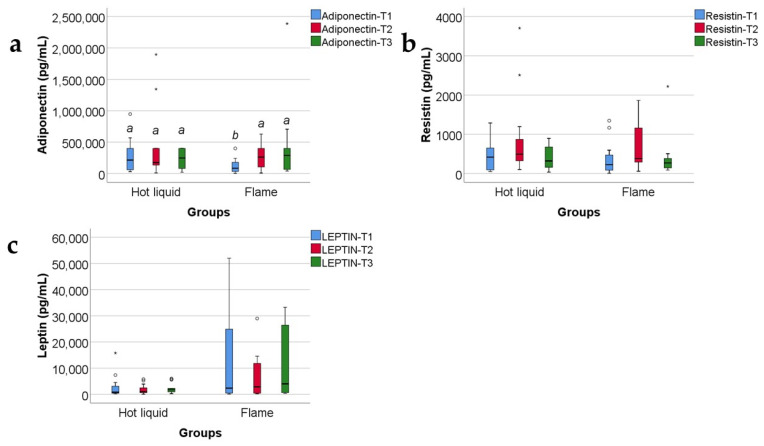
The three adipokines variations in the target group, as related to the mechanism of burn: hot fluid versus flame (concentrations, in pg/mL). Medians with IQR are depicted. Mann–Whitney U tests—statistical analysis. A significant difference between measurements is marked by unidentical lowercase letters. No statistically significant differences were observed for resistin, nor for leptin (*p* > 0.05). In the box-plot graphical representation, the ° symbol marks an outlier for a measurement < 1st quartile-1,5IQR or a measurement > 3rd quartile +1,5IQR. The * symbol marks an extreme outlier, for a measurement < 1st quartile-1,5IQR or a measurement > 3rd quartile +1,5IQR.

**Figure 17:**
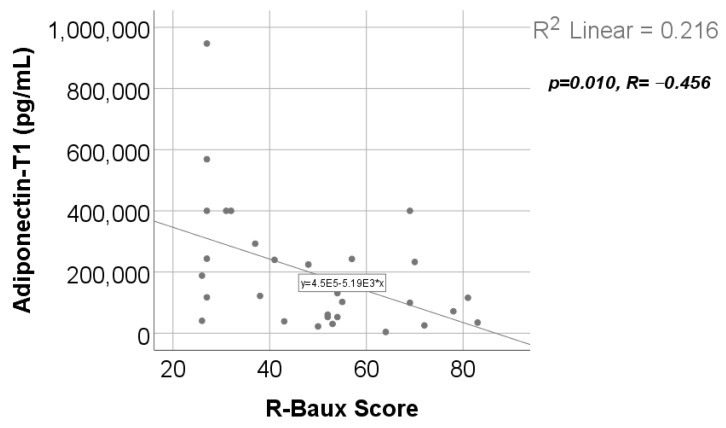
Correlation between R-Baux Score and adiponectin value at T1.

Decreased leptin serum levels at 10 days and 21 days correlated significantly with prolonged hospitalization period in our study (Table S10, Figure 15a,b). As a clinical application, the constant decrease in leptin serum values heralds prolonged hospitalization and worsening of the prognosis in severely burned children.

Leptin is the only adipokine from our study that, at 48 h and 10 days post burn, has a statistically significant and positive correlation with TNF-α (Tables S6 and S7 and Figure 5b and Figure 9a), which is considered one of the main pro-inflammatory cytokines in severe burns. This is concordant with other studies reporting increased leptin serum levels in severely burned children [65,67] and in other conditions involving a systemic inflammatory response or disturbances of the glucose and/or lipides metabolisms [68,69,70]. The other two studied adipokines (adiponectin and resistin) have a statistically significant but negative correlation with TNF-α at 10 days post burn, in our study. These observations underline the complex and different roles of adipokines in the biologic response in severe burns. We might conclude that, adiponectin, resistin, and leptin serum levels offer a good image of the intensity of the systemic inflammatory response in the first three weeks post burn, together with other markers of inflammation like CRP and TNF-α.

At 10 days post burn, increased leptin serum levels statistically significantly correlate with decreased PAI-1 serum levels (Table S8, Figure 9b). Other studies reported a positive correlation between leptin and PAI-1 serum levels in obesity and metabolic syndrome. The explanation was an upregulation of PAI-1 expression in vascular endothelial cells, induced by leptin [71,72].

### 3.4. Triglycerides

Triglycerides were determined in our study, because they were considered an indicator of fat cell lipolysis and also a marker of the peripheral resistance to the insulin’s antilipolytic effect [73]. TGL serum levels were statistically significantly higher at 21 days than at 10 days and at 48 h post burn, reflecting an accentuated process of peripheral fat cell lipolysis (Tables S4 and S5 and Figure 3d).

In our study, in severely burned children, increased TGL serum levels appear to correlate strongly with the systemic inflammatory response reflected by increased CRP and PAI-1 serum values (Table S8 and Figure 12b,c). Positive correlations between CRP and TGL serum levels were reported: (a) in patients with dyslipidemia and increased risk for atherosclerosis [74]; (b) in patients with accelerated progression of Alzheimer’s disease [75]; (c) in patients with type 2 diabetes mellitus and dyslipidemia [76], the common denominator being, in such patients, the systemic inflammatory state. Strong correlations between PAI-1 serum levels and TGL serum levels were reported in patients with dyslipidemia, obesity, metabolic syndrome, type 2 diabetes mellitus, and nonalcoholic fatty liver disease [77,78,79,80]. PAI-1 is considered to have an important role in the development of lipid profile alterations. DNAm PAI-1 (DNA methylation estimator of plasma PAI-1 levels) strongly predicts plasma lipid levels in type 2 diabetes mellitus, nonalcoholic fatty liver disease (NAFLD) and systemic hypertension [81].

In the end of the discussions section, we want to address study’s main limitations:

1. Single-center experience.
2. Rather limited volume of the target group (due to the COVID-19 pandemic).
3. Study conducted only three weeks post burn; lack of compliance for follow-up after this discharge.
4. Unidentical values of median age in the target group and the reference group.
  Perspectives for the following studies:
  - multi-center study;
  - both children and adult patients;
  - larger target group;
  - longer follow-up after discharge.

## 4. Materials and Methods

The same work protocol was presented in our previous paper [7]. 

### 4.1. Patients Included in this Research

An observational cohort prospective study was performed between 2022 and 2023. Using the following inclusion and exclusion criteria, we enrolled 32 children, from the burn unit of Grigore Alexandrescu Children’s Emergency Hospital in Bucharest. This study was performed after obtaining the Agreement of the Ethics Committee of Grigore Alexandrescu Hospital No. 28656/12.10.2021.

The present paper continues the publication of the results of the abovementioned study. We have already published another paper that fructifies the results of our study, concerning plasminogen activator inhibitor-1, α 1-acid glycoprotein, C-reactive protein, and platelet factor 4. 

### 4.2. Inclusion and Exclusion Criteria for the Children with a Burn (Study Group)

#### 4.2.1. Inclusion Criteria: Children with a Burn (Study Group)

- Age below 18 years old;
- Thermal burns involving at least 25% TBSA;
- Patients admitted to the burn unit in less than 48 h from the moment of burn infliction;
- The patient’s parents or legal guardian read, understood, and signed the informed consent that states their agreement for the enrolment of their child/children into the present study;
- Patient agreement to be part of this study.

#### 4.2.2. Exclusion Criteria: Children with a Burn (Study Group)

- Pre-existing autoimmune health condition;
- Local or systemic infection at the moment of admission into the burn unit;
- Pre-existing oncologic condition;
- Patients who have been receiving hormonal treatment;
- Patients who have been receiving oncologic treatment;
- Patients who have been receiving immunosuppressive therapy;
- Refusal of the parents or legal guardians to enroll the patient into the present study;
- Refusal of the patient to be included in this study.

### 4.3. Inclusion and Exclusion Criteria for the Control Group

#### 4.3.1. Inclusion Criteria: Control Group

- Age below 18 years old;
- The individual agrees to be included in the control group;
- The patient’s parents or legal guardians read, understood, and signed the informed consent that states their agreement for the enrolment of their child/children into the present study.

#### 4.3.2. Exclusion Criteria: Control Group

- Inflammatory systemic condition;
- Autoimmune health condition;
- Local or systemic infection;
- Oncologic condition;
- Individual under hormonal treatment, or oncologic treatment, or immunosuppressive therapy;
- Individuals with oral health conditions (teeth, gums, mucosa);
- Refusal of the parents or legal guardians to enroll the patient in the present study;
- Refusal of the individual to be part of this study.

After applying the criteria mentioned above, 21 subjects, from the pediatric ward of the same hospital, were included in the control group.

### 4.4. Sample Collection

#### 4.4.1. Sample Collection in the Study Group

For each patient, samples of blood were obtained at three different moments:

- Forty-eight hours after the burn trauma (T1);
- Ten days after the burn trauma (T2);
- Twenty-one days after the burn trauma (T3).

#### 4.4.2. Sample Collection in the Control Group

For the subjects in the control group, samples of blood were also obtained, during the hospitalization in the pediatric ward.

### 4.5. Sample Preservation

The samples were fast-frozen at −20 degrees Celsius in the abovementioned hospital. Then, the samples were transported on ice, in isotherm bags, and stored at −80 degrees Celsius in the Biochemistry laboratory freezer.

### 4.6. Sample Analyzing and Data Collecting Using Multiplex Technique

Adiponectin, resistin, and PAI-1 were detected using Milliplex human adipokine magnetic bead panel 1 (HADK1MAG-61K), while for TNF-α and leptin, we used Milliplex Human adipokine magnetic bead panel 2 (HADK2MAG-61K). Milliplex Human CVD panel 3 (acute phase) magnetic bead panel (HCVD3MAG-67-K) was used to detect CRP. All the kits were provided by Merck Group, the European branch (Darmstadt, Germany).

The PAI-1, CRP, adiponectin, resistin, leptin, and TNF-α (among another 9 biochemical parameters, not presented in this paper) were detected using the Multiplex technique. All the samples, standards, and controls were brought to room temperature and incubated with an assay buffer and specific beads overnight at 4 °C with shaking. After removing all the content from the wells and washing the plate three times, the detection antibody was added followed by a 1 h incubation. The streptavidin–phycoerythrin was added with a 30 min incubation. In the end, all the content from the wells was removed, the plate was washed three times again, sheath fluid was added, and the mean florescence intensity (MFI) was read on Luminex 200. CRP concentration was expressed in ng/mL. Adiponectin, resistin, PAI-1, leptin, and TNF-α concentrations were expressed in pg/mL. Triglycerides were detected during hospitalization using an automatic biochemistry analyzer, the concentration being expressed in mg/dL.

We collected several clinical and epidemiological types of data, which we retained for our study: TBSA, hospitalization period length, age, and burn mechanism. All the data collected from the children’s samples in the study group and from the subjects’ samples in the control group were anonymized.

### 4.7. Data Analysis

All the data from this study were analyzed using IBM SPSS Statistics 25 and illustrated using Microsoft Office Excel/Word 2021. Quantitative variables were tested for normal distribution using the Shapiro–Wilk test and were written as averages with standard deviations or medians with interquartile ranges. Quantitative variables were tested between measurements using Friedman’s tests along with Dunn–Bonferroni post hoc tests. Quantitative independent variables were tested between groups using Mann–Whitney U tests, and correlations between them were calculated using the Spearman’s rho correlation coefficient. Qualitative variables were written as absolute frequencies with percentages and were tested between groups using Fisher’s Exact Test.

## 5. Conclusions

Significant statistical differences were obtained for resistin and leptin compared to the control group, in different moments of measurements. Adiponectin serum levels at 48 h presented a statistically significant and positive correlation with hot liquid as mechanism of burn, and a statistically significant and negative correlation with the Revised Baux score and with TBSA. Adiponectin serum levels at 48 h, 10 days, and 21 days correlated statistically significantly and positively with resistin and PAI-1 serum levels. Other statistically significant and positive correlations were found between adiponectin and CRP at 10 and 21 days, adiponectin and leptin at 10 days, and adiponectin and TGL at 21 days. A statistically significant and negative correlation was found between adiponectin and TNF-α at 10 days post burn. Resistin serum levels presented statistically significant and positive correlations with adiponectin, CRP, and PAI-1 at 48 h, 10 days, and 21 days post burn. At 10 days, resistin serum levels presented a statistically significant and negative correlation with leptin and TNF-α. We found a statistically significant and negative correlation between the leptin serum levels at 10 days and at 21 days and the length of hospitalization. Leptin serum levels correlate positively and statistically significantly with TNF-α at 48 h and 10 days post burn. At 10 days, leptin correlates statistically significantly: and negative with resistin and with PAI-1; and positive with adiponectin. 

We might conclude that, in children with severe burns, each of the studied adipokines has a specific evolution of the serum levels, and all of them present particular statistically significant correlations with some of the clinical parameters (Revised Baux score, burn mechanism, length of hospitalization, TBSA) and with proteins involved in the systemic inflammatory response and into the hypermetabolic response.

## Figures and Tables

**Table 1:** Characteristics of the studied groups.

Group/Parameter	Study Group	Control Group	*p* *
Number of patients (Nr., %)	32 (60.4%)	21 (39.6%)	-
Gender—Male (Nr., %)	22 (68.8%)	9 (42.9%)	0.089 *
Age (Median (IQR)) (years)	3 (2–10)	14 (12–16)	<0.001**
Age (Median (IQR)) (months)	39.5 (26–122.5)		
Hospitalization period (Median (IQR))	56.5 (25.25–94)	-	-
TBSA (Median (IQR))	35 (30–45)	-	-
% Burned body surface-Burns grade IIB/III (Median (IQR))	20 (16.5–33)		
Time from event to hospitalization (Median (IQR))	8 (4–9.5)	-	-
R-Baux Score (Median (IQR))	50 (31–64)	-	-
*Burn injury mechanism* (Nr. %)		-	-
Hot liquid	15 (46.9%)	-	-
Flame	13 (40.6%)	-	-
Electric arc	4 (12.5%)	-	-
Usage of mechanical ventilation (Nr., %)	25 (78.1%)	-	-
Smoke inhalation injury (Nr., %)	11 (34.4%)	-	-
Mortality (Nr., %)	2 (6.3%)	-	-

* Fisher’s Exact Test, ** Mann–Whitney U Test.

## Data Availability

Data supporting the reported results are available from the authors.

## Supplementary Materials

The following supporting information can be downloaded at: https://www.mdpi.com/article/10.3390/ijms25147630/s1.
