# Supplementary material for: Adipokines—A Cohort Prospective Study in Children with Severe Burns
Source: Int J Mol Sci. 2024 Jul 11;25(14):7630. doi: 10.3390/ijms25147630 (PMC11277113; doi:10.3390/ijms25147630)
Supplement: Supplementary file 1 [file ijms-25-07630-s001.zip › ijms-3042132-supplementary.pdf]

Table S1. Evolution of analyzed adipokines in the study group

Measurement (Median (IQR))	T1	T2	T3	p*
**Adiponectin**	117369 (46597-243211)	238186 (105056-400000)	227977 (67597-400000)	0.104
**Resistin**	335.56 (95.25-622.3)	445.6 (317.62-1012)	267.5 (146.2-450.13)	**0.006**
**Leptin**	1558.9 (449.8-4508.5)	1233.7 (522-3898.4)	1885.3 (674.9-5885.4)	0.097

***Related-Samples Friedman's Two-Way Analysis of Variance by Ranks**

Table S2. Post-hoc comparison of resistin evolution in the study group

Measurement*	T1	T2	T3
**T1**	-	**0.012**	1.000
**T2**	**0.012**	-	**0.026**
**T3**	1.000	**0.026**	-

***Post-hoc Dunn-Bonferroni Test**

Table S3. Comparison of analyzed adipokines between the study and control group

Parameter/Group (Median (IQR))		T1	T2	T3
**Adiponectin**	**Control**	164518 (50825-400000)	164518 (50825-400000)	164518 (50825-400000)
	**Study**	119639 (43564-243479)	256206 (114188-400000)	227977 (67597-400000)
	**p***	0.453	0.381	0.503
**Resistin**	**Control**	188.38 (87.02-274.18)	188.38 (87.02-274.18)	188.38 (87.02-274.18)
	**Study**	316.36 (110.38-588.46)	445.6 (316.8-915.89)	267.5 (146.2-450.13)
	**p***	0.052	**<0.001**	0.053
**Leptin**	**Control**	3476.16 (1171.2-10705.8)	3476.16 (1171.2-10705.8)	3476.16 (1171.2-10705.8)
	**Study**	2313.7 (482.7-4785.6)	1357.4 (610.2-3550.5)	1871.76 (693.8-5790.26)
	**p***	0.119	**0.041**	0.166

***Mann-Whitney U Test**

Table S4. Evolution of usual serum parameters in the study group

Parameter (Median (IQR))	T1	T2	T3	p*
**CRP**	2.86 (1.54-6.82)	4.26 (1.73-7.21)	2.02 (0.93-4.12)	**0.014**
**TNF-alpha**	13.24 (6.24-42.6)	12.22 (7.65-31.1)	13.35 (8.75-71.86)	**0.009**
**PAI-1**	132.81 (40.09-277.3)	231.75 (106-536.65)	177.77 (83.02-297.13)	0.055
**Triglycerides**	102.5 (78-125.75)	111 (88.5-141)	134 (92.25-173.75)	**0.002**

***Related-Samples Friedman's Two-Way Analysis of Variance by Ranks**

Table S5. Post-hoc comparison of parameters evolution in the study group

*CRP**	**T1**	**T2**	**T3**
**T1**	-	0.881	0.198
**T2**	0.881	-	**0.012**
**T3**	0.198	**0.012**	-
*TNF-alpha**	**T1**	**T2**	**T3**
**T1**	-	1.000	**0.019**
**T2**	1.000	-	**0.029**
**T3**	**0.019**	**0.029**	-
*Triglycerides**	**T1**	**T2**	**T3**
**T1**	-	1.000	**0.003**
**T2**	1.000	-	**0.040**
**T3**	**0.003**	**0.040**	-

***Post-hoc Dunn-Bonferroni Test**

Table S6. Correlation matrix for analyzed parameters at T1

**Parameter***	**Adiponectin**	**Resistin**	**Leptin**	**CRP**	**TNF- α**	**PAI-1**	**TGL**
**Adiponectin**	-	**p<0.001**<br>**R=0.680**	p=0.175<br>R= -0.254	**p=0.007**<br>**R=0.470**	p=0.811<br>R=0.046	**p<0.001**<br>**R=0.621**	p=0.482<br>R=0.129
**Resistin**	**p<0.001**<br>**R=0.680**	-	p=0.075<br>R= -0.330	**p<0.001**<br>**R=0.665**	p=0.928<br>R=0.017	**p<0.001**<br>**R=0.776**	p=0.547<br>R= -0.111
**Leptin**	p=0.175<br>R= -0.254	p=0.075<br>R= -0.330	-	p=0.500<br>R= -0.128	**p=0.036**<br>**R=0.384**	p=0.167<br>R= -0.259	p=0.739<br>R= -0.063
**CRP**	**p=0.007**<br>**R=0.470**	**p<0.001**<br>**R=0.665**	p=0.500<br>R= -0.128	-	p=0.087<br>R=0.318	**p<0.001**<br>**R=0.604**	p=0.570<br>R= -0.104
**TNF- α**	p=0.811<br>R=0.046	p=0.928<br>R=0.017	**p=0.036**<br>**R=0.384**	p=0.087<br>R=0.318	-	p=0.890<br>R= -0.026	p=0.871<br>R=0.031
**PAI-1**	**p<0.001**<br>**R=0.621**	**p<0.001**<br>**R=0.776**	p=0.167<br>R= -0.259	**p<0.001**<br>**R=0.604**	p=0.890<br>R= -0.026	-	p=0.285<br>R= -0.195
**TGL**	p=0.482<br>R=0.129	p=0.547<br>R= -0.111	p=0.739<br>R= -0.063	p=0.570<br>R= -0.104	p=0.871<br>R=0.031	p=0.285<br>R= -0.195	-

***Spearman's rho Correlation Coefficient, TGL = Triglycerides**

Table S7. Correlation matrix for analyzed parameters at T2

Parameter*	Adiponectin	Resistin	Leptin	CRP	TNF- α	PAI-1	TGL
**Adiponectin**	-	**p=0.001 R=0.560**	**p=0.035 R= -0.379**	p=0.105 R=0.297	**p=0.012 R= -0.447**	**p<0.001 R=0.699**	p=0.733 R= -0.066
**Resistin**	**p=0.001 R=0.560**	-	**p=0.024 R= -0.404**	**p=0.005 R=0.490**	**p=0.001 R= -0.572**	**p<0.001 R=0.805**	p=0.780 R= -0.054
**Leptin**	**p=0.035 R= -0.379**	**p=0.024 R= -0.404**	-	p=0.814 R=0.044	**p=0.018 R=0.423**	**p=0.017 R= -0.425**	p=0.737 R= -0.065
**CRP**	p=0.105 R=0.297	**p=0.005 R=0.490**	p=0.814 R=0.044	-	p=0.433 R= -0.146	p=0.093 R=0.307	p=0.743 R=0.064
**TNF- α**	**p=0.012 R= -0.447**	**p=0.001 R= -0.572**	**p=0.018 R=0.423**	p=0.433 R= -0.146	-	**p<0.001 R= -0.715**	p=0.832 R=0.041
**PAI-1**	**p<0.001 R=0.699**	**p<0.001 R=0.805**	**p=0.017 R= -0.425**	p=0.093 R=0.307	**p<0.001 R= -0.715**	-	p=0.847 R=0.037
**TGL**	p=0.733 R= -0.066	p=0.780 R= -0.054	p=0.737 R= -0.065	p=0.743 R=0.064	p=0.832 R=0.041	p=0.847 R=0.037	-

***Spearman's rho Correlation Coefficient, TGL = Triglycerides**

Table S8. Correlation matrix for analyzed parameters at T3

Parameter*	Adiponectin	Resistin	Leptin	CRP	TNF- α	PAI-1	TGL
**Adiponectin**	-	**p=0.015 R=0.447**	p=0.596 R= -0.103	p=0.528 R=0.122	p=0.563 R=0.112	**p<0.001 R=0.774**	**p=0.039 R=0.392**
**Resistin**	**p=0.015 R=0.447**	-	p=0.558 R= -0.114	**p=0.011 R=0.465**	p=0.250 R=0.221	**p<0.001 R=0.640**	p=0.060 R=0.359
**Leptin**	p=0.596 R= -0.103	p=0.558 R= -0.114	-	p=0.193 R=0.249	p=0.072 R=0.339	p=0.726 R=0.068	p=0.171 R=0.266
**CRP**	p=0.528 R=0.122	**p=0.011 R=0.465**	p=0.193 R=0.249	-	p=0.078 R=0.333	p=0.251 R=0.220	**p<0.001 R=0.615**
**TNF- α**	p=0.563 R=0.112	p=0.250 R=0.221	p=0.072 R=0.339	p=0.078 R=0.333	-	p=0.929 R= -0.017	p=0.073 R=0.344
**PAI-1**	**p<0.001 R=0.774**	**p<0.001 R=0.640**	p=0.726 R=0.068	p=0.251 R=0.220	p=0.929 R= -0.017	-	**p=0.015 R=0.457**
**TGL**	**p=0.039 R=0.392**	p=0.060 R=0.359	p=0.171 R=0.266	**p<0.001 R=0.615**	p=0.073 R=0.344	**p=0.015 R=0.457**	-

***Spearman's rho Correlation Coefficient, TGL = Triglycerides**

Table S9. Correlations between adipokines and TBSA

Correlation*/Parameter	T1	T2	T3
**Adiponectin x TBSA**	**p=0.018, R= -0.416**	p=0.600, R= -0.098	p=0.074, R= -0.337
**Resistin x TBSA**	p=0.101, R= -0.295	p=0.672, R= -0.079	p=0.788, R= -0.052
**Leptin x TBSA**	p=0.767, R= -0.057	p=0.658, R= -0.083	p=0.681, R= 0.080

***Spearman's rho Correlation Coefficient**

Table S10. Correlations between adipokines and the hospitalization period

Correlation*/Parameter	T1	T2	T3
**Adiponectin x Hospitalization**	p=0.732, R= -0.063	p=0.262, R= 0.208	p=0.573, R= -0.109
**Resistin x Hospitalization**	p=0.870, R= -0.030	p=0.595, R= 0.099	p=0.860, R= 0.034
**Leptin x Hospitalization**	p=0.101, R= -0.306	**p=0.021, R= -0.412**	**p=0.035, R= -0.393**

***Spearman's rho Correlation Coefficient**

Table S11. Comparison of adipokines between patients according to the burn injury mechanism

Parameter/Mechanism (Median (IQR))		T1	T2	T3
**Adiponectin**	**Hot liquid**	239812 (60634-400000)	186467 (135323-400000)	247032 (79956-400000)
	**Flame**	71495 (27929-177394)	263152 (105056-400000)	288931 (64302-400000)
	**p***	**0.019**	0.964	0.860
**Resistin**	**Hot liquid**	388.7 (94.16-649.9)	445.61 (198.12-871.19)	322.6 (144.9-685.57)
	**Flame**	226.18 (85.84-471.78)	416.37 (292.11-1160.85)	269.82 (142.74-389)
	**p***	0.525	1.000	0.667
**Leptin**	**Hot liquid**	804.45 (389.37-3325)	1121.93 (610.2-2796.6)	1733.25 (831-3097)
	**Flame**	2747.48 (449.8-24872.5)	2692.4 (782.75-9765.44)	1759.6 (684.3-21622.6)
	**p***	0.507	0.201	0.494

***Mann-Whitney U Test**

Table S12. Correlations between adipokines and R-Baux Score

Correlation*/Parameter	T1	T2	T3
**Adiponectin x Score**	**p=0.010, R= -0.456**	p=0.669, R= -0.081	p=0.543, R= -0.120
**Resistin x Score**	p=0.513, R= -0.122	p=0.535, R= -0.118	p=0.624, R= -0.097
**Leptin x Score**	p=0.973, R= -0.007	p=0.861, R= 0.033	p=0.478, R =0.140

***Spearman's rho Correlation Coefficient**
